# Cargo specificity, regulation, and therapeutic potential of cytoplasmic dynein

**DOI:** 10.1038/s12276-024-01200-7

**Published:** 2024-04-01

**Authors:** Jin-Gyeong Park, Hanul Jeon, Kwang Yeon Hwang, Sun-Shin Cha, Rafael T. Han, Hyesung Cho, In-Gyun Lee

**Affiliations:** 1 Biomedical Research Division, Korea Institute of Science and Technology, Seoul, 02792 South Korea; 2 Department of Biotechnology, College of Life Sciences and Biotechnology, Korea University, Seoul, 02841 South Korea; 3 Department of Chemistry & Nanoscience, Ewha Womans University, Seoul, 03760 South Korea; 4 KHU-KIST Department of Converging Science and Technology, Kyunghee University, Seoul, 02447 South Korea; 5 Extreme Materials Research Center, Korea Institute of Science and Technology, Seoul, 02792 South Korea; 6 Department of Biological Chemistry, University of Science and Technology, Daejeon, 34113 South Korea

**Keywords:** Dynein, Single-molecule biophysics

## Abstract

Intracellular retrograde transport in eukaryotic cells relies exclusively on the molecular motor cytoplasmic dynein 1. Unlike its counterpart, kinesin, dynein has a single isoform, which raises questions about its cargo specificity and regulatory mechanisms. The precision of dynein-mediated cargo transport is governed by a multitude of factors, including temperature, phosphorylation, the microtubule track, and interactions with a family of activating adaptor proteins. Activating adaptors are of particular importance because they not only activate the unidirectional motility of the motor but also connect a diverse array of cargoes with the dynein motor. Therefore, it is unsurprising that dysregulation of the dynein-activating adaptor transport machinery can lead to diseases such as spinal muscular atrophy, lower extremity, and dominant. Here, we discuss dynein motor motility within cells and in in vitro, and we present several methodologies employed to track the motion of the motor. We highlight several newly identified activating adaptors and their roles in regulating dynein. Finally, we explore the potential therapeutic applications of manipulating dynein transport to address diseases linked to dynein malfunction.

## Dynein Motor Motility: New Adaptors Unlock Disease Treatment Potential

This research examines how parts move within a specific type of cell, called eukaryotic cells, focusing on the role of a component called cytoplasmic dynein 1 in moving various items. However, understanding dynein’s function is difficult due to its complexity and the systems that control it. Recent progress in structural, biophysical (relating to the physical properties of biological molecules), and cellular methods are helping us understand these processes. The research also looks at dynein’s role in human diseases, especially neurodegenerative diseases, and the possibility of using this knowledge for treatment. The scientists emphasize the need for more research into dynein’s molecular structure and the potential treatment possibilities this research could offer.

This summary was initially drafted using artificial intelligence, then revised and fact-checked by the author.

## Introduction

Within the cytoplasm of eukaryotic cells, various functionally necessary cellular components undergo continuous movement from one location to another. Long-range transport is accomplished by the movement of molecular motors along the microtubule (MT) track, while myosin movement along actin filaments provides short-range transport^1–3^. Cytoplasmic dynein 1 (hereafter referred to as dynein) is responsible for transporting a wide array of diverse cargoes along the MT track and performing numerous functions within eukaryotic cells^4,5^. In contrast to microtubule-based anterograde motor kinesin transport cargoes, which exhibit diverse isoforms for cargo binding, dynein has a single isoform^6,7^. This observation raises questions regarding how cargo specificity is achieved and how regulatory mechanisms govern dynein-mediated intracellular transport^8^. Elucidating the mechanisms that govern the cargo specificity of dynein has been a longstanding focus of research on dynein function. Nonetheless, due to the immense complexity and dynamic nature of the molecule, systematic investigations of the dynein motor in cellular contexts as well as in noncellular models in vitro have been very challenging, making it difficult to achieve a comprehensive understanding of the molecular mechanism of the motor^9,10^. Owing to recent advances in structural, biophysical, and cellular approaches, we are now gaining insight into the intricate processes by which dynein facilitates the intracellular transport of various cargoes.

Given the complexity of the dynein molecule and its involvement in critical cellular functions, of the involvement of a variety of regulatory factors in dynein-mediated cargo transport is no surprise^4,5,11^. The cargo specificity, activity and force of the dynein motor depend on multiple intramolecular and intermolecular factors, including temperature^12^, phosphorylation^13,14^, the MT track^15–17^, and “activating adaptor” binding^11^. In particular, the family of dynein activating adaptors, of which new members continue to be discovered, is among the major regulators governing the cargo specificity of the dynein motor as well as its force and velocity^11,18,19^. An activating adaptor not only binds directly to the dynein–dynactin complex and stabilizes it in the active conformation but also links various cargoes to the motor and activates the motility of the motor. Considering the importance of activating adaptors in dynein regulation, mutations affecting the functionality of an activating adaptor or perturbing interactions between dynein and an activating adaptor can result in malfunctions of the dynein complex^20,21^. Consequently, these mutations are associated with a range of human diseases, notably neurological pathologies such as spinal muscular atrophy, Charcot–Marie–Tooth disease, cortical development malformations, and neurodegenerative diseases^22–24^. Here, we discuss the motility of the motor dynein motor in cells and in noncellular models in vitro and explore the methodologies employed to track the movement of dynein in both contexts. We then describe several recently identified activating adaptors and explore the potential of leveraging the dynein transport machinery for therapeutic applications.

## Cytoplasmic dynein 1 transport machinery

**Fig. 1:**
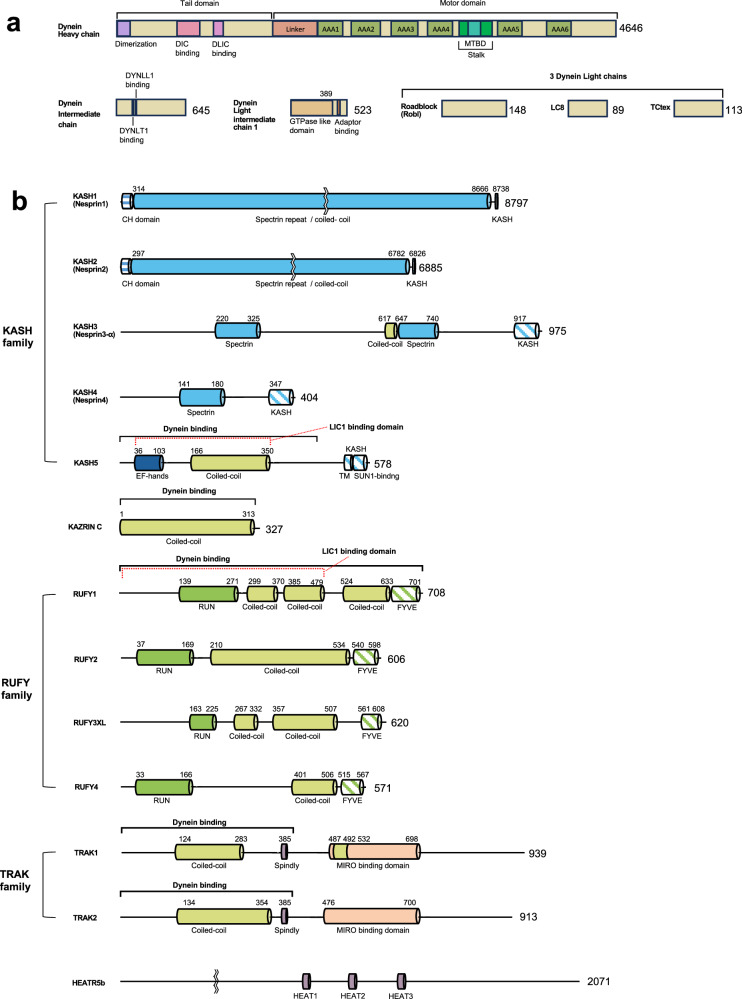
Domain architecture of the dynein complex and several recently identified dynein adaptors. **a** Domain architecture of the dynein heavy chain, intermediate chain, light intermediate chain 1, and three light chains. Note that the representation of the three light chains is not to scale. **b** Domain architecture of several recently identified dynein adaptors and their families.

Two types of cytoplasmic dynein are responsible for the retrograde transport of cargoes along the MT track within the cytoplasm (cytoplasmic dynein 1) and within cilia and flagella (cytoplasmic dynein 2; intraflagellar transport [IFT] dynein)^7,25^. Here, we focus on cytoplasmic dynein 1 and refer to it as “dynein”. Human dynein consists of six distinct polypeptide chains, namely, the heavy chain (HC), the intermediate chain (IC), the light intermediate chain (LIC), and three types of light chains (roadblock [Robl], LC8, and Tctex)^25^ (Fig. 1a). Each of these constituents is present in duplicate, culminating in the formation of a complex with an approximate molecular mass of approximately 1.4 MDa^25^. By itself, mammalian dynein lacks the capability to selectively engage with particular cargoes and is incapable of processive movement^26,27^. To undergo processive movement along the MT track in coordination with specific cargo, mammalian dynein forms a complex with two additional components^11,28,29^: (i) the general adaptor dynactin and (ii) a family of coiled-coil proteins known as “activating adaptors” that recruit specific cargoes to the dynein complex, facilitating the processive motility of the complex. The assembly of the ~3.5 MDa dynein–dynactin-adaptor (DDA) complex introduces a considerable level of complexity and heterogeneity. Therefore, systematically investigating the function, structure, interactions, and molecular mechanisms of this motor both within cells and in noncellular models in vitro is very challenging, often necessitating specialized techniques, as discussed below.

## Tracking dynein-driven motility in living cells

The principal techniques for visualizing dynein complexes within cells involve the direct labeling of dynein subunits using synthetic fluorophores or recombinant fluorescent proteins such as green fluorescent protein (GFP)^30–35^ (Table 1). Although directly labeling dynein with a fluorescent marker allows the observation of dynein motility and behavior within a cell, the direct high-resolution visualization of dynein movement in living cells often remains challenging. Many cargoes are attached to other motors and exhibit bidirectional movement along the MT track, precluding the direct determination of dynein-driven motility. For example, in living *Dictyostelium discoideum* cells, dynein moves at a speed of ~1.8 µm/s toward the minus end of the MT and ~1.7 µm/s toward the plus end of the MT^30^, implying that both the minus-end-directed motor (e.g., dynein) and the plus-end-directed motor (e.g., kinesins) are simultaneously associated with a certain cargo and that a regulatory mechanism exists to coordinate the activity of the bound motors to control the direction and velocity of cargo transport. Consistent with this, in mammalian cells, GFP-labeled dynein moves along the MT track in both plus end- and minus end-directed motion with a velocity of 1–2 µm/s^34^. Furthermore, as dynein is ubiquitously expressed at high levels throughout the cytoplasm of many cells, it is often challenging to distinguish dynein moving along the MT track from the diffuse background of dynein in the cytoplasm.

**Table 1:** Several strategies for observing dynein motility in cells.

Observation technique	Labeling strategy	Characteristic properties	Refs.
Fluorescence microscopy (in *Dictyostelium* cells)	Dynein IC-GFP fusion	Bidirectional movement	^30^
Fluorescence microscopy (in COS cells)	Viral glycoprotein VSVG -GFP fusion	ER–Golgi transport	^31^
Fluorescence microscopy (in *Aspergillus nidulans* cells)	Dynein HC-GFP fusion	Colocalization with moving endosome.	^32^
Fluorescence microscopy (in *Ustilago maydis* cells)	Dynein HC- 3x GFP fusion	Accumulates at MT plus ends	^33^
Fluorescence microscopy (in HeLa cells)	Dynein IC2/HC/LIC1-GFP fusion	Bidirectional movement	^34^
Fluorescence microscopy (in hippocampal neurons)	Dynein IC-GFP fusion (Knock-in mouse)	Anterograde movement of dynein mediated by kinesin 1	^35^
HILO microscopy	Dynein HC-GFP fusion	Bidirectional movement	^38^
HILO microscopy	Dynein HC-Halo fusion (Endogenously labeled)	Long range movement in the axon	^39^

*IC* intermediate chain, *HC* heavy chain, *LIC* light intermediate chain, *VSVG* vesicular stomatitis virus G protein, *ER* endoplasmic reticulum, *MT* microtubule, *TIRF* total internal reflection fluorescence microscopy, *HILO* highly inclined and laminated optical sheet.

These challenges can at least be partly overcome by imaging and tracking dynein movement using advanced superresolution microscopy techniques, such as highly inclined and laminated optical sheet (HILO) and minimal photon flux (MINFLUX) microscopy, which can enable the observation of more detailed features of dynein movement. HILO microscopy generates a thin excitation plane of several microns^36^, increasing the signal/background ratio and decreasing photobleaching compared with conventional confocal microscopy because of the nonfocused illumination, thereby providing better spatial and temporal resolution and enabling near-single-molecule tracking of the motor protein inside living cells^37^. When the movement of mouse dynein heavy chain labeled with GFP in HeLa cells was tracked via HILO microscopy, approximately 30% of the dynein bound to MTs showed processive movements toward the minus end of the MT over a relatively short period (~0.5 s), and the average length was less than one micron^38^. This finding implies that when traveling distances greater than one micron, dynein–dynactin–cargo adaptor complexes consistently undergo binding and unbinding. While overexpressed GFP–dynein shows a short residence time and short run length inside the cytoplasm of HeLa cells, dynein shows much more robust movement and a longer run length along the axons of human neuronal cells^39^. Endogenously labeled dynein had a much longer run length of up to ~110 μm and was able to move the entire length of the axon, with an average speed of ~1.7 μm/s, as observed via HILO microscopy. These observations suggest that dynein-driven transport can differ according to cell type and cellular environment, including factors such as the arrangement and dynamics of MT tracks^39^. Intriguingly, an additional superresolution technique known as MINFLUX microscopy has demonstrated the ability to achieve very high localization precision of a fluorophore, detecting as few as ~20 photons^40,41^. This technique has been successfully utilized to track the movement of kinesins with high spatial and temporal resolution in cells. Utilizing MINFLUX, the movement of kinesin motors in live cells can be pinpointed with sub-millisecond temporal resolution and a spatial resolution of approximately one nanometer^42,43^. While this approach has been applied to investigate kinesin behavior within living cells, it has yet to be extended to the study of dynein.

Instead of directly observation of the movement of fluorescently labeled dynein in cells, the motion of dynein can be assessed by tethering the complex to nonnative, static cargoes and subsequently measuring the movement of the cargo instead of that of the motor complex^44,45^ (Table 2). Inducible cargo trafficking assays artificially recruit the motor complex to a specific cellular compartment (e.g., stationary vesicles such as peroxisomes) using a heterodimerization system such as FKBP-rapalog-FRB^34,46,47^. The heterodimerization of FKBP and the FKBP–rapamycin binding domain (FRB) is triggered by the addition of a cell-permeable, non-immunosuppressive analog of rapamycin called rapalog. Additionally, a photoactivation system that uses light to achieve noninvasive and high spatiotemporal resolution has emerged as an important system for studying dynein motility in cells^34,45,48,49^. In this case, the cTMP-Halo tag (cTMP-Htag), which consists of a Halo-tag ligand linked to a photocaged trimethoprim (TMP), is used as a dimerizer. This molecule enables the heterodimerization of Halo tag proteins (Halo) and *Escherichia coli* (*E. coli*) dihydrofolate reductase (eDHFR) upon the cleavage of photocaged cTMP–Htag by 405-nm light. When the motor protein is tagged with eDHFR and model cargo vesicles (such as peroxisomes) are tagged with Halo, 405-nm light can induce motor-specific vesicle movement^48,49^ (Table 2).

**Table 2:** Examples of inducible cargo trafficking assays tracking dynein motility in cells.

Methodology	Dynein label	Vesicle label	Refs.
Chemically induced dimerization (Dimer pair: FKBP-FRB; dimerizer: rapalog)	BICD2 (dynein adaptor)-FRB	FKBP-GFP-Rab6 vesicle	^34^
	Dynein HC-FRB	FKBP-RFP-Peroxisome	^44^
Photoactivatable dimerization (Dimer pair: eDHFR-Halo; dimerizer: cTMP-Htag)	BICD2 (dynein adaptor) - mCherry-eDHFR	GFP-Halo-Peroxisome	^48^
	Hook1 (dynein adaptor) -mCherry-eDHFR	GFP-Halo-Peroxisome	^49^
	Hook3 (dynein adaptor)-mCherry-eDHFR		

*FKBP* FK506-binding protein, *FRB* FKBP-rapamycin binding protein, *DHFR* dihydrofolate reductase, *HC* heavy chain.

Compared to direct dynein subunit labeling strategies, inducible cargo trafficking assays enable the observation of motor-specific translocation events in a more controlled spatiotemporal context. In inducible cargo trafficking assays, dynein-driven motility can be examined by live-cell imaging via high-resolution total internal reflection fluorescence (TIRF) microscopy to analyze particle distribution and velocity. Compared to the bidirectional and stochastic movement of subunit-labeled dynein, vesicles recruited to dynein through the heterodimerization system exhibited unidirectional motility along the MT track, with a mean velocity of ~1 µm/s.

## Tracking dynein-driven motility in vitro

By reconstituting the dynein–dynactin complex and adaptor protein, the motility of the dynein complex can be directly observed in a much more controlled context, excluding many cellular environmental factors that affect the motility of the motor. The greatest pitfall of this system is the purification of extremely complex, multisubunit components (i.e., dynein, dynactin, and the activating adaptor) and the reconstitution of the active massive tripartite motor. Each component can be purified separately and then reconstituted^50,51^ or can be purified from brain tissue or mammalian cells using a purified activating adaptor as bait^28,52^. Otherwise, cellular lysates containing overexpressed fluorescently labeled adaptors can be directly used for imaging without a purification step^49^, although this method cannot reliably exclude other factors (e.g., other adaptor complexes) that might affect the motility of the motor. The motility of the motor can then be observed using total internal reflection microscopy.

## Dynein-activating adaptors

The motility and function of mammalian dynein depend on a wide variety of factors, with one critical regulatory factor being its interaction with the activating adaptor family^11^. Dynein forms a tripartite complex with its general adaptor dynactin and the activating adaptor, and this complex can achieve robust motility on MT tracks^18,53–56^. The term “activating adaptor” is more specific than the general terms “adaptor” and “cargo adaptor”. This distinction arises from experimental evidence that activating adaptors not only link cargo to dynein-like adaptors but also enhance the processive motion of the dynein motor^11^. Since the discovery of activating adaptors that facilitate the motility of the dynein complex, extensive research has been conducted on various activating adaptor families, revealing their roles in regulating motor velocity, cargo recognition, and force generation. The activating adaptor family generally performs dual functions: (i) releasing the autoinhibited conformation (and stabilizing the activated conformation) of dynein through interaction with its N-terminal LIC-binding domain and the very long (~200–300 amino acids) central coiled-coil domain that can run along the whole dynactin filament and ii) linking the specific cargo to the dynein–dynactin complex through its cargo-binding domain at the C-terminus^11^. Although the activating adaptor families share no sequence homology, these common features enable the categorization of those families as activating adaptors. Different activating adaptors have different effects on the activation^57^ of the dynein complex. This variation is evident in the differences in both velocity and run length within the dynein–dynactin–adaptor (DDA) complex, which differ depending on the specific type of activating adaptor that is attached.

At the time of writing, more than a dozen activating adaptors (e.g., Hook1, Hook3, BICD2, BICDR1, Spindly, NIN (Ninein), NINL (Ninein-like), CRACR2a, Rab45, Rab11-FIP3 (FIP3), KASH5, TRAK1, TRAK2, and JIP3) have been identified^28,49,57–69^. Given the existence of comprehensive review papers on activating adaptors^11,70^, we offer a concise overview of several recently identified activating adaptors as well as potential candidates in the following section (Fig. 1b and Table 3).

**Table 3:** Several recently identified dynein (activating) adaptors.

Adaptor	Evidence of LIC1 binding	Evidence of dynein-dynactin motility activation	Cellular cargo	Localization	Refs.
KASH5	Pull down, ITC	Single-molecule imaging with TIRF (with purified proteins)	Chromosome	Nuclear envelope	^57,62^
Kazrin C	Pull down	Not been directly tested	Early endosome	Pericentriolar region	^67^
RUFY1	Pull down	FKBP-FRB heterodimerization assay (indirect evidence)	Recycling endosome	Golgi, TGN	^68^
TRAK1/2	Single-molecule imaging with TIRF (indirect evidence)	Single-molecule imaging with TIRF (with lysate and purified proteins)	Mitochondria	Outer mitochondrial membrane	^63,90^
HEATR5B	N/A	N/A (unlikely)	AP1-positive endosomal membranes	AP1-positive structures in the cytoplasm	^69^

*LIC1* light intermediate chain 1, *ITC* isothermal titration calorimetry, *TIRF* total internal reflection fluorescence, *TGN* trans-Golgi network, *AP1* adaptor protein-1.

### KASH5

Dynein plays a crucial role during cell division^71^. Its critical functions include chromosome movement and segregation, centrosome maturation and separation, and proper positioning and maturation of the mitotic spindle^72,73^. To achieve chromosome movement during cell division before the nuclear envelope breaks down, dynein tethers to the chromosome by interacting with the linker of the nucleo- and cytoskeleton (LINC) complex located in the nuclear envelope^74^. The LINC complex connects the cytoskeleton, including microtubules, with the nucleus, transmitting the forces generated by the dynein complex to the chromosome^74^.

The LINC complex spans the double membrane of the nucleus. The core components of the LINC complex are the highly conserved Sad1/UNC-84 (SUN) protein, which spans the inner nuclear membrane, and the Klarsicht/ANC-1/SYNE homology (KASH) protein^75^. The KASH protein typically comprises spectrin repeats or coiled-coil domains, which extent from the outer nuclear membrane to the cytoplasm, and a single transmembrane domain followed by an ~30 aa KASH domain that interacts with the SUN protein in the space between the outer and inner nuclear membranes^76^.

During prophase I of meiosis, KASH5, a meiosis-specific isoform of KASH, interacts with SUN1 in the perinuclear space, forming a prophase I-specific LINC complex that links dynein to the chromosomes inside the nuclear membrane^77^. KASH5 immunoprecipitates with dynein and shares common structural features with activating adaptors, such as N-terminal EF-hands followed by coiled coils comprising ~200 amino acids^77^. This observation led to the hypothesis that KASH5 functions as an activating adaptor. Recent studies have provided experimental evidence supporting this idea, demonstrating that KASH5 binds directly to the LIC subunit of dynein, forms a complex with dynein–dynactin, and thereby activates its motility^57,62^. These observations confirmed KASH5 as the first known activating adaptor containing a transmembrane domain. Given that other KASH proteins also contain LIC binding domains at the N-terminus followed by long coiled coils, it would be intriguing to explore whether other KASH isoforms also serve as activating adaptors^78^.

### Kazrin C

Kazrin is an evolutionarily conserved cytoplasmic protein that is widely expressed in vertebrates^79^. However, it does not exhibit significant sequence homology with other known proteins, and its exact function has not been fully elucidated. Kazrin has been reported to have various functions, including desmosome assembly, cell adhesion, cytoskeleton organization, and epidermal differentiation^79,80^. In particular, among the seven isoforms (A-F and K), Kazrin C is specifically involved in early endosome (EE) trafficking, as it attaches directly to several EE components through its C-terminal intrinsically disordered region (IDR)^67^. Knocking out Kazrin C or inhibiting dynein disrupts the colocalization of juxtanuclear localization EEs and Kazrin C, suggesting that Kazrin C might be involved in dynein-mediated retrograde transport. The domain architecture of Kazrin C is also similar to that of other dynein-activating adaptors, as it contains an N-terminal globular domain, followed by long coiled coils and C-terminal IDRs that interact with vesicular compartments. Furthermore, Kazrin C interacts directly with the LIC1 subunit of dynein, further suggesting that Kazrin C might be a new member of the family of dynein-activating adaptors^67^. Although Kazrin C has not been directly tested for the ability to function as an activating adaptor (i.e., single-molecule motility assay using purified components), it would be intriguing to determine its role in dynein-mediated endosomal trafficking.

### RUFY1

RUFY proteins are a family of cytosolic proteins that contain an N-terminal RUN domain (named after the proteins RPIP8, UNC-14 and NESCA), a C-terminal FYVE domain (named after the proteins Fab1, YOTB/ZK632.12, Vac1, and EEA1), and coiled-coil domains. The RUN and FYVE domains define the characteristic molecular features of the RUFY protein family, which comprises four members in mammals: RUFY1, RUFY2, RUFY3, and RUFY4^81^. The FYVE domain specifically binds to the membrane-embedded phosphatidylinositol 3-phosphate (PI3P) through its zinc finger domain and consecutively targets the RUFY proteins toward the endosomal membrane^82^. The RUN domain at the N-terminus has been shown to interact with diverse GTPases^83^ and is thus involved in GTPase signaling^84^. Since the domain architecture of the RUFY family resembles that of the dynein activating adaptor family and since RUFYs participate in intracellular cargo trafficking, vesicular transport and fusion, RUFYs have been hypothesized to t function as activating adaptors of dynein.

Recently, RUFY1 was shown to interact directly with Arl8b, an Arf-like small GTPase protein^68,85^. Arl8b is a member of the ARF (ADP-ribosylation factor) family, which is a subgroup of the small GTPase superfamily. Like other small GTPase proteins, Arl8b is involved in various cellular processes, including intracellular vesicle trafficking, lysosomal function, and endosomal dynamics^86^. RUFY1 binds directly to Arl8b through its RUN domain at the N-terminus and colocalizes with Arl8b to Rab14-positive recycling/sorting endosomes, suggesting a collaborative role of RUFY1 and Arl8b in orchestrating endosomal trafficking processes^68^. Notably, proteomic investigation revealed interaction between RUFY1 and the dynein/dynactin subunits. A pull-down analysis further confirmed the direct interaction between the RUN domain of RUFY1 and the LIC1 subunit of the dynein complex, which is one of the common characteristics of dynein-activating adaptors^68^.

In addition to RUFY1, RUFY3 and RUFY4 have been shown to interact directly with Arl8b^85^. Compared to RUFY1, which participates in endosomal sorting in cells, RUFY3 and RUFY4 colocalize in lysosomes and participate in lysosomal positioning. Akin to RUFY1, RUFY3, and RUFY4 also immunoprecipitated with dynein/dynactin subunits; furthermore, purified RUFY3 directly interacted with the LIC1 subunit of the dynein complex. Targeting RUFY3 and RUFY4 to the stationary vesicle peroxisome promoted perinuclear clustering in a dynein- and dynactin-dependent manner, further supporting the hypothesis that RUFY3 and RUFY4 might function as dynein-activating adaptors^85^.

### TRAK1/2

The bidirectional transport of mitochondria along microtubules is achieved by dynein and kinesin^87^. This process involves precise coordination between the mitochondrial adaptor Mitochondrial Rho-like (Miro) and the motor adaptor Trafficking Kinesin protein (TRAK)^88^. Miro, a Rho GTPase protein embedded in outer membrane of mitochondria via its C-terminal transmembrane domain, interacts with the dynein adaptor TRAK through its N-terminal tandem EF-hand pair ligand mimic (ELM) domain^89,90^. Both TRAK isoforms 1 and 2 feature a long coiled-coil structure at their N-terminus that recruits the dynein–dynactin complex. Additionally, this region mediates interactions with the opposing motor kinesin^64^, thereby facilitating the bidirectional movement of mitochondria. Recent single-molecule imaging using purified proteins as well as cell lysates demonstrated that TRAK can activate mammalian dynein–dynactin, confirming its role as a dynein-activating adaptor^63,90^.

### HEATR5B

Due to the lack of sequence homology among cargo adaptor families, identifying cargo adaptors based solely on amino acid sequences is often challenging. Accordingly, a proteomic approach has been employed to identify dynein–dynactin cargo adaptors, leading to the identification of several novel families of activating adaptors^60^. HEAT repeat-containing protein 5B (HEATR5B) was recently identified as a dynein tail interactor in a proteomic search using the dynein tail domain (comprising dynein heavy chain residues 1-1079, intermediate chain, light intermediate chain, and 3 light chains) as bait^69^. HEATR5B is a component of the clathrin-coated vesicle (CCV) machinery, suggesting that this protein is involved in vesicle trafficking in the trans-Golgi network (TGN). In the TGN, HEATR5B interacts with adaptor protein complex-1 (AP-1), which coordinates cargo selection and CCV formation. In HeLa cells, HEATR5B comigrated with AP-1-positive structures and promoted the membrane localization and motility of AP-1-positive structures, suggesting that HEATR5B not only acts as a dynein/dynactin binder but also increases the motility of the dynein–dynactin complex^69^. Although HEATR5B can promote the motility of the dynein–dynactin complex in cells, it seems likely that HEATR5B is not a canonical activating adaptor, as the protein lacks long coiled coils that can run along the dynactin filament and mediate interaction with the dynein tail. Instead, HEATR5B might act as a scaffold for all dynein–dynactin–cargo adaptors, analogous to Ankyrin B^91^.

## Dynein as a drug target

Dynein has been implicated in numerous diseases, particularly those affecting the neurological system^92^. Given the elongated shape of neurons, the disruption of microtubule-based transport resulting from mutations or dysfunction of components of the dynein motor complex can lead to neuronal degeneration and ultimately to various human neurological disorders^92–96^. Indeed, mutations in the cytoplasmic dynein 1 heavy chain (DYNC1H1)-encoding gene have been implicated in neurological disorders such as spinal muscular atrophy, lower extremity, dominant (SMA-LED)^22^, Charcot–Marie–Tooth disease, axonal, type 2 O (CMT2O)^23^, and malformations of cortical development (MCD)^24^. Among the array of potentially disease-associated mutations that can be found in DYNC1H1, those correlated with SMA-LED are predominantly localized to the dynein tail domain (Table 4). The tail domain of the dynein heavy chain is responsible for homodimerization as well as interactions with dynactin, other subunits, and activating adaptors^25^. Interestingly, mutations in genes encoding BICD2, a representative and well-studied cargo adaptor, have also been implicated in SMA-LED^97–103^. This observation implies that the relationship between dynein and BICD2 might play an important role in the pathophysiology of SMA-LED.

**Table 4:** Examples of disease-related dynein heavy chain mutations.

Protein variation in tail domain	N199S [119], L196S [111], R251C [112], R251L [113], R264G [114, 115], D338N [115], R399G [115], M581L [115], I584L [115], R598L [115], R598C [115], E603V [115], I609T [117], V612M [115], W673C [115], G807S [118], Y970C [115], D1062G [119,110], V1116A [120], Q1194R [121], H1412 [120], P1511L [120].
Protein variation in motor domain	R1962H [122], E2616K [115], E3048K [121], S3360G [119,110], G3658E [120].

Spinal muscular atrophy, lower extremity, dominant (SMA-LED) is a very rare subtype of spinal muscular atrophy (SMA), which is a diverse group of human neurodegenerative genetic disorders characterized by the degeneration of spinal motor neurons^104,105^. Generally, the degeneration of motor neurons leads to skeletal muscle weakness and atrophy (wasting), particularly in the muscles closest to the center of the body, such as those in the back, shoulders, hips, and thighs. SMA encompasses various forms, each of which presents with shared characteristics but a distinct genetic profile, often impacting specific subsets of neurons and muscles^106^. SMA can generally be categorized by pattern of weakness, severity, progression of symptoms, mode of inheritance, and associated mutations. The most common form of SMA (type I SMA or autosomal recessive proximal SMA) is caused by the loss of the *survival motor neuron 1* (*SMN1*) gene encoding the protein SMN1, which is essential for motor neuron survival^106^. SMA-LED represents an exceedingly rare subtype of SMA, initially identified in a North American family^107^. Unlike the majority of SMA types, which are recessively inherited, SMA-LED is inherited in a dominant manner^105^. SMA-LED patients exhibit prominent quadriceps atrophy and weakness of hip adductors, with normal upper extremity muscle strength and sensation and without cognitive retardation^108^. SMA-LED can be further divided into SMALED1 and SMALED2, which are caused by heterozygous mutations in the dynein 1 heavy chain (*DYNC1H1*) and the activating adaptor *BICD2*, respectively^109^.

While numerous disease-related mutations have been documented^110–121^, the specific pathological impacts of the *DYNC1H1* or *BICD2* mutations underlying SMALED1 and SMALED2 have not been determined. In an in vitro single-molecule motility assay, the dynein–dynactin–BICD2 (DDB) complex with SMA-LED1-associated mutations in *DYNC1H1* was shown to decrease the number of processive complexes, the run length, the velocity^20^. In contrast, the disease-associated mutation in *BICD2* increases the interaction between dynein and BICD2^52^. Furthermore, compared with wild-type BICD2, the increased binding results in a significantly elevated quantity of motile dynein molecules, suggesting that disease-associated mutations in BICD2 hyperactivate the DDB complex^52^. In addition to affecting the functionality of the dynein motor transport machinery, disease-associated mutations in *BICD2* have other functional consequences that cause pathological defects. For example, overexpression of the BICD2 gene in primary motor neurons has been shown to increase the stability of the MT track, accompanied by axonal aberrations^122^. Importantly, mutations linked to neuropathy within the kinesin motor affect motor activity, underscoring the essential need for a proper balance of motor function in both the anterograde and retrograde directions for optimal neuronal health^123–127^.

The role of dynein in human disease has prompted increasing interest in developing small-molecule dynein modulators for investigating the functional mechanisms of dynein and exploring the possibility of mitigating diseases associated with dynein^128–132^ (Table 5). The characteristics of these small-molecule compounds, including their binding sites and functional consequences, vary greatly. Given the critical role of dynein in many human diseases, modulating disease-specific dynein-associated malfunctions using a small-molecule compound would be of great interest.

**Table 5:** Several small molecules that inhibit dynein activity.

Inhibitor	Mechanism of dynein inhibition	Initial screening method	Refs.
Ciliobrevin	Inhibition of ATPase activity (ATP competition)	Hedgehog signaling assay	^128^
Dynapyrazole	Inhibition of ATPase activity (ATP competition)	Chemical structure-based analysis (isostere of ciliobrevin)	^129^
Compounds 19/20	Disruption of allosteric communication between AAAs (by AAA3/AAA4 binding)	Derivatives of ciliobrevin/dynapyrazole	^130^
Dynarrestin	Decoupling of ATP hydrolysis from MT binding cycle	Motor neuron differentiation assay	^131^

### Prospects

The intricate nature of the dynein molecule has hampered the comprehensive understanding of the molecular mechanisms governing dynein regulation. Nonetheless, recent advances in structural, biophysical, and superresolution microscopy techniques are enabling the elucidation of how this massive and complex molecule functions in such a diverse range of tasks.

Considering the diversity of the functional properties of dynein, including those related to cargo transport, it is reasonable to speculate that a broader array of adaptor families may exist for the specific purpose of linking diverse cargoes to the dynein molecule, as well as controlling the velocity and force generation of the molecule. Given the central importance of dynein in cellular physiology, especially within neuronal contexts, unraveling the molecular foundations of dynein-mediated cargo transport holds significant therapeutic potential.
